# Antibody-Based Strategies to Prevent and Treat Influenza

**DOI:** 10.3389/fimmu.2015.00315

**Published:** 2015-07-13

**Authors:** Zachary Shriver, Jose M. Trevejo, Ram Sasisekharan

**Affiliations:** ^1^Visterra Inc., Cambridge, MA, USA; ^2^Department of Biological Engineering, Koch Institute of Integrative Cancer Research, Massachusetts Institute of Technology, Cambridge, MA, USA; ^3^Infectious Diseases Interdisciplinary Research Group, Singapore-MIT Alliance for Research and Technology, Singapore, Singapore

**Keywords:** influenza A virus, monoclonal antibodies, mutations, therapeutics, hemagglutinins, viral

## Abstract

Passive immunization using antibodies is a promising alternative to other antiviral treatment options. The potential for seasonal protection arising from a single injection of antibodies is appealing and has been pursued for a number of infectious agents. However, until recently, antibody-based strategies to combat infectious agents have been hampered due to the fact that most antibodies have been found to be strain specific, with the virus evolving resistance in many cases. The discovery of broadly neutralizing antibodies (bNAbs) in influenza, dengue virus, and HIV, which bind to multiple, structurally diverse strains, has provided renewed interest in this area. This review will focus on new technologies that enable the discovery of bNAbs, the challenges and opportunities of immunotherapies as an important addition to existing antiviral therapy, and the role of antibody discovery in informing rational vaccine discovery – with agents targeting influenza specifically addressed. Multiple candidates have entered the clinic and raise the possibility that a single antibody or small combination of antibodies can effectively neutralize a wide variety of strains. However, challenges remain – including combating escape variants, pharmacodynamics of antibody distribution, and development of efficacy biomarkers beyond virologic endpoints.

## Introduction

New alternative countermeasures for influenza are urgently needed. Vaccines to seasonal and pandemic influenza are foundational to provide widespread herd immunity to influenza. However, most inactivated and live-attenuated vaccine technologies are strain specific – requiring constant updating of the strains used in yearly multivalent vaccine preparations. In addition, severe influenza disease occurs each season in many high risk groups, to whom the vaccine provides limited or no protection, such as young children, the elderly, patients that are immunocompromised or who have pulmonary conditions, inflammatory conditions, or malignancies, as well as pregnant women (1, 2). In addition to vaccines, current treatment and prophylaxis of influenza is limited to the neuraminidase inhibitors oseltamivir (Tamiflu) and zanamivir (Relenza). Despite the availability of these treatments, 10–44% of hospitalized patients require intensive care and 25–50% of these patients die. In the United States, it is estimated that over 200,000 patients are hospitalized with influenza, with up to 48,000 deaths, per year (3). A comparison of annual mortality rates from infectious disease in the United States further demonstrates the lack of effective interventions against this deadly disease (Table S1 in Supplementary Material).

Furthermore, certain strains of influenza have resulted in infections with high mortality rates: the 1918 H1N1 Spanish Flu strain resulted in deaths of 1–3% of the world’s population, compared to the 1968 pandemic strain that resulted in the death of 0.03% of the world’s population (4). More recently, Avian H5 strains have had documented mortality rates up to 60% despite the use of currently licensed anti-viral treatments (5). Finally, the continued emergence of resistance to current anti-viral drugs increases the need for new therapeutics (6).

## Passive Immunotherapy has a Long History

Prior to the advent of antibiotics, convalescent serum was the only antidote available for bacterial toxins, such as diphtheria and tetanus (7). Eventually, it was discovered that the protective properties within the serum were predominantly due to neutralizing antibodies. The therapeutic use of passive antibodies has been well established for several viral infections. There are licensed polyclonal antibody products for several viruses, including hepatitis B (HBIG), varicella (VZIG), cytomegalovirus (Cytogam), rabies (HRIG), and respiratory syncytial virus (RSV) (Respigam). More recently, monoclonal antibodies (mAbs) for viral infections have been developed, including licensure of Synagis for prevention of RSV infection. mAbs for the prevention and treatment of a number of other viral targets, including Hepatitis C, Rabies, and West Nile Virus, are in clinical development (8–11). Historically, these antibody products have primarily focused on the prevention of viral disease, although application to treatment of infection has been investigated for RSV (12). Of note, no monoclonal antibody-based solution has been approved for the *treatment* of active infection.

In the absence of development of a universal, broadly protective vaccine, passive immunization using antibodies potentially offers several benefits. First, passive immunity provides the opportunity to protect at-risk individuals from infection. At-risk segments of the population include first responders to a relatively novel strain as well as those who do not mount an immune response to vaccines including the immunocompromised, those in poor health, pregnant women, and critically ill patients. Indeed, recent modeling analyses completed by us and our collaborators (M. Boni, Oxford University Clinical Research Unit) indicate that for a sufficiently potent and long-lasting antibody (*t*_1/2_ = ~18 days, protective period = 3 half-lives), population coverage of only 4–6% would be required to significantly reduce hospitalization rates. Notably, given the current state-of-the-art in the production of antibodies, it is possible to readily generate enough monoclonal antibody to protect the population at the epicenter of an epidemic outbreak in a much shorter time scale than that of vaccine production (>6 months) (13). The potential for long-lasting protection, covering an entire exposure period (i.e., an entire season for influenza), arising from a single injection of antibodies is appealing and has been pursued for a number of infectious agents. For example, in the case of hepatitis A, prophylactic administration of immunoglobulins can protect against viral exposure. Additionally, post-exposure prophylaxis with immunoglobulin is >85% effective in preventing hepatitis A if administered within 2 weeks after viral exposure, and efficacy is even higher when administered early in the incubation period (14).

The use of antibody therapy to treat influenza has recently received more attention, with some clinical experience to support efficacy. A meta-analysis of studies conducted during the 1918 pandemic using blood products strongly supports a benefit for treated patients (15). Overall, the six studies documented a 21% reduced risk of mortality in treatment groups (16 vs. 37% mortality in controls, 95% of risk difference, CI: 15–27). Furthermore, a recent study evaluated the use of convalescent plasma in 93 patients with H1N1 2009 influenza in Hong Kong (16). In this prospective multi-center case-control study, patients with severe influenza, who were hospitalized and required intensive care unit support, were recruited and offered convalescent plasma containing influenza neutralizing antibody in addition to the standard of care with either oseltamivir or zanamivir. Mortality was significantly lower in the treatment groups who received convalescent plasma compared to the controls (20.0 vs. 54.8%, *p* = 0.01).

Given the overall promise (and current limitations) of passive immunization approaches, as well as the overarching goal of accounting for viral mutations, it is highly desirable to identify and/or engineer antibodies that bind with high affinity and that neutralize many or all strains that are capable of infecting humans. To this end, there is intense focus on antibodies that bind to influenza hemagglutinin (HA) since antibodies to HA are known to be protective in animal models and in humans. The challenge is that HA is a highly diverse protein; there are 18 subtypes categorized into two groups – 1 and 2. As such, the ability to identify an antibody or small collection of antibodies that can bind to and neutralize all clinically relevant strains is a substantial challenge. The ability to rapidly identify broadly neutralizing antibodies (bNAbs) has been enhanced by the development of several new high-throughput technologies that now promise to enable comprehensive tracking of all of the immunological cell subsets, extending even to the level of the individual clones of B cells that carry out adaptive responses (17, 18). Improvements in the toolkit for human immunological studies are continually evolving and are likely to increase our understanding/discovery of antibodies for therapeutic use.

Characterization of the overall B-cell response to infection or vaccination has provided potentially important insights into lasting immunity, including the heterogeneous nature of individualistic responses to vaccination/infection. However, with next-generation deep sequencing data, it has become clear that, in different individuals, expansion of B-cell clones in response to infection arising from similar or “convergent” antibody gene rearrangements can be detected. For example, tracking of B-cell clones following pandemic single antigen H1N1 vaccination revealed a strong clonal signature dominated by antibodies using the IGHV3-7V gene, and correspondence of highly similar CDR3 sequences in different humans. Convergent monoclonal antibody sequences display HA inhibition activity against H1N1 and other influenza strains (19). This raises the possibility of a so-called “universal” vaccine strategy-through selection of the appropriate immunogen(s) to elicit the most effective immune response. Other recent work using regions of or chimeric proteins derived from the stem region of HA seem to elicit a broadly protective response against multiple subtypes of influenza (20, 21). Furthermore, studies in the area of HIV have suggested that a broadly protective response may, in principle, be completed through eliciting specific B-cell responses (i.e., “training the immune system”) using temporally spaced immunization with different antigens (22).

In addition to the use of the above tools to study the adaptive immune response, there has recently been a concerted effort to identify, engineer, and characterize antibodies that bind to a number of influenza subtypes (so-called “broadly” neutralizing antibodies). Several of these antibodies are listed in Table 1. These broadly neutralizing mAbs are a new, promising modality for treatment of influenza, potentially across all strains of the virus. Such antibodies have been identified through panning the B-cell repertoire of vaccinated or infected individuals (23, 24) and are estimated to be ~0.001–0.01% of the total antibody response (23). Identification of such antibodies has generated interest for several reasons, including (i) mapping of the epitopes to which these antibodies bind provides insights into epitopes that can be targeted for vaccine development and (ii) the antibodies by themselves are useful products to provide passive immunization or therapeutic efficacy against a wide variety of influenza subtypes.

**Table 1:** **Recent discoveries in broadly neutralizing antibodies to influenza**.

Antibody	Target	Breadth	Development
CR6261	Stem region/HA	Group 1	Phase II
CR8020	Stem region/HA	Group 2	Phase II
CR9114	Stem region/HA	Group 1/group 2	Pre-clinical
F10	Stem region/HA	Group 1	Pre-clinical
F16	Stem region/HA	Group 1/group 2	Pre-clinical
TCN-032	M2	Group 1/group 2	Phase II
MHAA4549A	Stem region/HA	Group 1/group 2	Phase II
CH65	Receptor binding site/HA	H1	Pre-clinical
VIS410	Stem region/HA	Group 1/group 2	Phase II

## Study of bNAbs has Led to Insight on Universal Vaccine Targets

In the context of vaccine efforts, identification of bNAbs to infectious agents provides a basis for the design of more protective vaccine strategies (25). Recent work on the evolution of bNAbs containing the V_h_1-69 heavy chain demonstrates that somatic mutations to the germline, which result in recognition of a hydrophobic patch on the HA stem results in the antibodies becoming more hydrophobic and binding influenza HA with higher avidity (26). Another recent study characterized ~200 anti-stem antibodies and identified two key elements that are required for the initial development of most V_H_1-69 antibodies: a polymorphic germline-encoded phenylalanine at position 54 and a conserved tyrosine at position 98 in the third complementary determining region of the heavy chain (27). By tracing the development of such antibodies, these studies have demonstrated that it may be possible to develop an immunofocusing strategy to promote the production of bNAbs containing V_h_1-69.

## Properties of bNAbs

Antibodies to two major surface antigens, the M ion channel and HA, have been studied as potential passive immunotherapies (Table 1). Antibodies that target the highly conserved M2 protein possess the requisite breadth of binding across group 1 and group 2 (i.e., all subtypes of influenza A). However, such antibodies are non-neutralizing; the predominant mechanism of action for M2-specific antibody is indirect through ADCC-mediated killing of infected cells. This leads to incomplete protection. For example, in a lethal influenza mouse model; an M2-targeted antibody product required three injections with M2-specific antibodies at 24, 72, and 120 h post-infection and still provided only a partial (~60%) protective response (28).

In contrast, antibodies to HA can clearly neutralize influenza virus *in vitro*, provide complete protection after a single administration *in vivo*, and protect against multiple strains of influenza (24, 29, 30). Additionally, use of such antibodies likely also mitigates bacterial secondary infection, since rapid reduction in viral titers prevents bacterial adherence (31). These data are supported by the fact that the efficacy of current vaccine approaches to influenza (especially inactivated virus strategies) is measured by the HA neutralizing titer. However, through the processes of antigenic drift and shift, the HA of influenza virus can develop resistance to antibodies that target HA. Such has been the case with, for example, CR6261 (32), CR8020 (24), and F10 (33), where several mutations are known to lower the binding affinity of the antibody to HA and confer resistance. The fact that HA is subject to mutation and the virus can undergo reassortment questions whether an immunotherapy strategy can be adequately developed due to facile development of escape mutants.

## Strategies to Design Effective Immunotherapy

**Figure 1:**
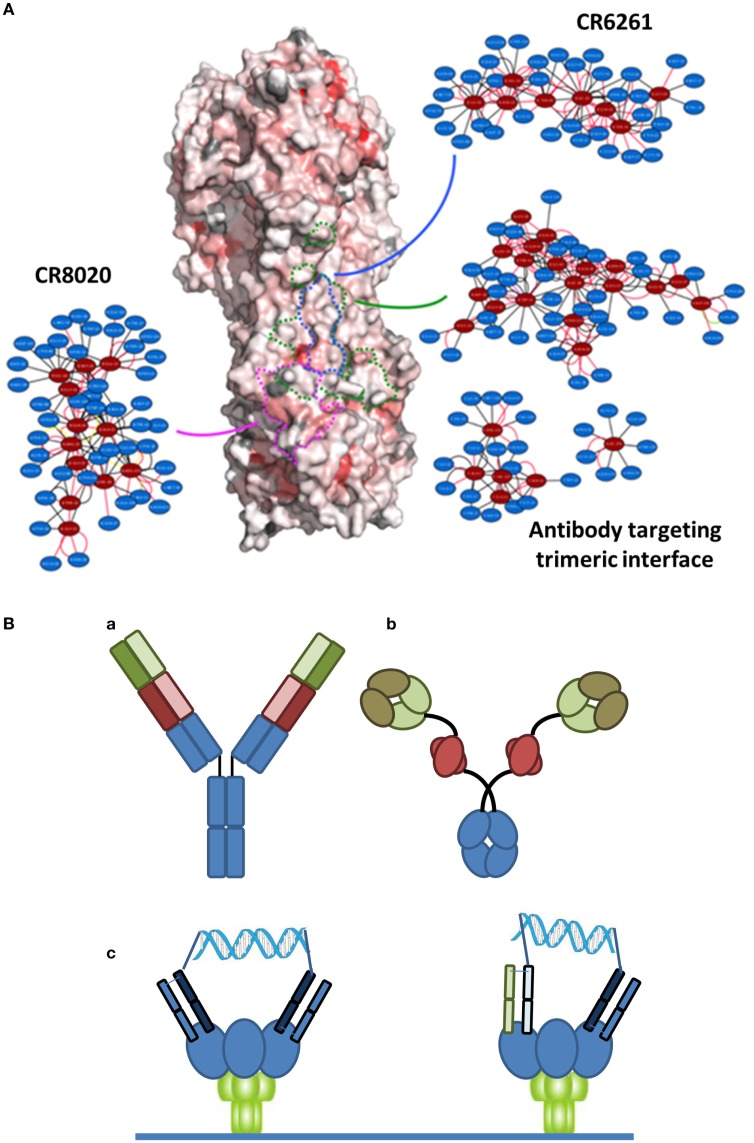
**(A)** Network-view of bNAb epitopes. HA trimer is represented in a solvent accessible surface format and colored based on normalized residue network scores. Coloring varies from white to red where white indicates poorly networked residues and red indicates highly networked residues. The three bNAb epitopes are highlighted by dotted borderlines (green: antibody targeting trimeric interface; blue: CR6261; pink: CR8020). The 2D network map of the epitope is also shown. A network is made up of nodes and edges. Nodes colored in red indicate functional epitope residues whereas nodes colored in blue indicate residues that are in the network environment of the epitope residues. **(B)** Different bispecific formats that have demonstrated activity against infectious disease targets. (a) A dual-variable domain immunoglobulin format containing two distinct Vh-Vl pairings (one in red and one in green) has demonstrated activity against hepatitis B. (b) A bispecific format where a single chain variable region against Psl (red) targets the antibody to the cell surface of *Pseudomonas* enables engagement of a traditional Vh-Vl paratope with the rarer PcrV target. (c) Crosslinking of binding domains of variable and constant regions (V_H_-CH_1_/V_L_-CL; Fabs), either homotypic (left) or heterotypic (right) with a defined DNA-based spacer enables more potent neutralization of HIV virus.

There are at least two points that need to be considered with regard to an ideal immunotherapeutic strategy, particularly when considering a variable system like HA. The first is the epitope targeted by the antibody. Many of the bNAbs in Table 1 target the relatively conserved stem region of HA. While certain stem-binding-antibodies target epitopes that can mutate under selective pressure with apparently little or no fitness cost, other epitopes are less amenable to mutation and are more likely to engender a fitness cost (34). A structure-based network approach (35) can be used to provide insights into the tolerance to mutability of an amino acid in a protein system. This approach is based on analysis of sequences across different viral surface proteins that reveal amino acids that are highly networked (higher weighted contacts with neighboring amino acids), and therefore are more constrained in their ability to mutate (Figure 1A). By targeting these amino acids, it is possible to generate an antibody-based solution that is more refractory to resistance development while still maintaining binding to a potent and broadly neutralizing epitope. Additionally, in the context of therapy, it is likely that any antibody can be used in combination with a neuraminidase inhibitor, where there appears to be synergistic activities due to distinct mechanisms of action (36). Furthermore, as has become apparent in other viral diseases, such as HIV or hepatitis C, combination approaches are less likely to elicit resistance. Finally, recent studies have indicated that the activity of bNAbs is enhanced in the presence of the natural immune response (37).

The second important point to an ideal strategy is that there are a number of engineering-based strategies outside of the variable or binding region that can be used to enhance the efficacy of the antibody-based solutions. Certainly, one of the relevant approaches is Fc engineering to enhance the recruitment of complement and/or innate immune cells. In the context of bNAbs against influenza, it is known that the various stem-binding antibodies are able to recruit complement and that Fc effector functions are critical to their protective effect (38). However, the efficiency of complement recruitment is based on the geometry of engagement, with some antibodies being able to better engage complement as compared to others (39). The effector functions can be further enhanced through engineering of Fc mutations and/or alteration of the glycosylation site to enhance ADCC. Finally, in other therapeutic areas, particularly oncology, there has been an emphasis on discovery of bispecific antibody formats (40) (Figure 1B). In the context of antibodies to infectious agents, initial examples have provided intriguing results. Alternative formats have been investigated including use of multiple antibody binding domains (V_H_-V_L_), or inclusion of antibody-like binding domains, such as scFv or Fab fragments. Recently, data have been reported for a bispecific antibody-like construct to *Pseudomonas* where one binding site binds to a high density ligand (Psl) and thus targets the antibody, and the other binding site targets a highly neutralizing epitope (PcrV) (41). Furthermore, with reference to viral diseases, a recent report of a bispecific antibody to hepatitis B reported synergistic activity compared to the activity of the parent antibodies alone (42). Finally, in HIV, where the density of the gp140 spike protein at the viral surface is highly limiting, bridging through the use of a bispecific antibody resulted in much higher activity (43). Such a dual-targeting strategy may also be useful for other viruses such as influenza.

## Conclusion

If technologies can identify high affinity, bNAbs, passive immunization can likely provide an important adjunctive prophylactic and therapeutic option to supplement vaccination technologies. Antibody-based therapies are generally safe and well-tolerated, particularly when the antigen is an exogenous target. Even one of the more common effects of therapy, which is the development of anti-drug antibodies, at most serves to limit drug exposure rather than resulting in significant adverse effects. Recent maturation of several tools in antibody characterization, discovery, and engineering may enable a resurgence of passive immunotherapy strategies. With several antibody candidates that are currently in clinical development for influenza (Table 1) and potentially others, it is likely that we will determine in the near future whether an old idea becomes a new powerful tool to counteract the rapidly evolving threat of influenza and other virus infections.

## Conflict of Interest Statement

The authors declare that the research was conducted in the absence of any commercial or financial relationships that could be construed as a potential conflict of interest.

## Supplementary Material

The Supplementary Material for this article can be found online at http://journal.frontiersin.org/article/10.3389/fimmu.2015.00315
