# Supplementary material for: Antibody-Based Strategies to Prevent and Treat Influenza
Source: Front Immunol. 2015 Jul 13;6:315. doi: 10.3389/fimmu.2015.00315 (PMC4500096; doi:10.3389/fimmu.2015.00315)
Supplement: Supplementary file 1 [file Table_1.PDF]

Supplementary Material

Antibody-Based Strategies to Prevent and Treat Influenza

Zachary Shriver, Jose M. Trevejo & Ram Sasisekharan*

*** Correspondence:** Ram Sasisekharan: rams@mit.edu

1. Supplementary Table

Table S1. US Mortality Rates from Top Infectious Agents

Disease	Approximate Deaths (Year)
Influenza	3-49,000 (yearly)
Methicillin-resistant *Staphlococcus aureus* (MRSA)	11,300 (2009)
Hepatitis C	15,000 (2007)
Acute immunodeficiency syndrome from HIV	12,700 (2007)
Pneumococcal disease from *Streptococcus pneumoniae*	5,000 (2009)
Hepatitis B	1,800 (2007)
